# Characteristics of Veterans Experiencing Homelessness using Telehealth for Primary Care Before and After COVID-19 Pandemic Onset

**DOI:** 10.1007/s11606-023-08462-3

**Published:** 2024-01-22

**Authors:** Lucinda B. Leung, Eunice Zhang, Karen Chu, Caroline Yoo, Sonya Gabrielian, Claudia Der-Martirosian

**Affiliations:** 1 Center for the Study of Healthcare Innovation, Implementation & Policy, Department of Veterans Affairs Greater Los Angeles Healthcare System, Los Angeles, CA USA; 2 Division of General Internal Medicine-Health Services Research, David Geffen School of Medicine, University of California Los Angeles, Los Angeles, CA USA; 3 Department of Psychiatry and Biobehavioral Sciences, David Geffen School of Medicine, University of California Los Angeles, Los Angeles, CA USA; 4 Division of General Internal Medicine, UCLA David Geffen School of Medicine/Department of Veterans Affairs Greater Los Angeles Healthcare System, Los Angeles, CA USA; 5 Veterans Emergency Management Evaluation Center, Department of Veterans Affairs, North Hills, CA USA; 6 Department of Veterans Affairs Desert Pacific Mental Illness Research, Education, and Clinical Center, Los Angeles, CA USA

**Keywords:** telehealth, primary care, homelessness, veterans

## Abstract

**Background:**

The COVID-19 pandemic expanded telehealth use across healthcare systems, including the Veterans Health Administration (VA). Little is known about how large-scale telehealth rollout affected access to primary care for patients experiencing homelessness.

**Objective:**

To examine the extent to which homeless-experienced veterans used telehealth services in primary care and to characterize users before and after the onset of the COVID-19 pandemic.

**Design:**

Retrospective cohort study, 3/16/2019–3/15/2022.

**Participants:**

394,731 veterans with homelessness diagnoses nationally using 4,068,109 primary care visits.

**Main Measures:**

The outcomes were use of 1 + telehealth visits (video, phone, secure messaging) for primary care during each year. Through multivariable regression models, we examined associations between telehealth use, patient characteristics (e.g., age, sex, race-ethnicity, comorbidity), and VA homeless services use (e.g., homeless-tailored primary care (HPACT), permanent supportive housing).

**Key Results:**

Compared to pre-pandemic, telehealth in primary care among homeless-experienced veterans increased substantially 2 years post-pandemic (video: 1.37% versus 20.56%, phone: 60.74% versus 76.58%). Secure messaging was low over time (1.57–2.63%). In adjusted models, video users were more likely to be young (65 + years: OR = 0.43, CI: 0.42–0.44), women (OR = 1.74, CI: 1.70–1.78), Black (OR = 1.14, CI: 1.12–1.16), Hispanic (OR = 1.34, CI: 1.30–1.38), and with more comorbidities (2 + on the Charlson Comorbidity Index; OR = 1.16, CI: 1.14–1.19), compared to video non-users. HPACT patients were less likely to use video (OR = 0.68, CI: 0.66–0.71) than other primary care patients. This was not observed among users of other VA homeless services.

**Conclusions:**

Despite decreased access to health information technology and low pre-pandemic telehealth use, veterans experiencing homelessness still sustained high use of telehealth in primary care post-pandemic. Women and racial-ethnic minorities had higher video uptake proportionately, suggesting that telehealth may address access disparities among these homeless-experienced patient groups. Identifying and targeting organizational characteristics (e.g., HPACT users) that predict telehealth use for improvement may be key to increasing adoption among VA primary care patients experiencing homelessness.

**Supplementary Information:**

The online version contains supplementary material available at 10.1007/s11606-023-08462-3.

## INTRODUCTION

The COVID-19 pandemic expanded the availability of telehealth care options, but many patients face challenges using virtual modalities to access essential primary care services.^1^ Patients are now routinely allowed to communicate with primary care teams using several telehealth modalities, including phone, video, and secure messaging.^2^ Yet, there exists a “Digital Divide,” that is unequal digital health literacy and access to technology (e.g., smartphones, tablets, laptops) or internet required for patients to participate in telehealth care.^3^ As virtual care increased out of necessity, researchers documented telehealth disparities by age, income, race-ethnicity, etc., as well as differing patient preferences for its use.^3–8^ Many have studied how to optimally incorporate telehealth modalities into existing primary care models for at-risk demographic groups.^9, 10^ Homeless-experienced persons often fall into several of these at-risk categories and may thus be disproportionately affected by the Digital Divide.

Historically, persons experiencing homelessness have faced technological barriers (e.g., lack of mobile telephones) in accessing services and require supportive outreach to engage in primary care.^11–13^ Small-scale studies have documented attempts to engage homeless-experienced persons using telehealth care, as well as their perspectives regarding this care modality.^14, 15^ At the same time, telehealth has shown promise and is a tool that may reduce racial-ethnic and other disparities in chronic disease care over time.^16^ Common examples of telehealth modalities used by primary care teams include conducting outreach phone calls or responding to patient secure messages. Video telehealth adoption, however, has and continues to be low in primary care, as compared to specialty care.^6^ Observations during the COVID-19 pandemic may offer insights about large-scale telehealth adoption, as well as predictive characteristics of telehealth users, among individuals experiencing homelessness.

In response to the COVID-19 pandemic, the Veterans Health Administration (VA) made telehealth broadly available for all VA primary care patients, including veterans experiencing homelessness.^17^ The VA has invested heavily in housing and ensuring accessible health care services for more than 33,000 veterans who experience homelessness on any given night across the nation.^18^ VA funds a breadth of services for veterans with homeless experiences, including outreach for unsheltered veterans (e.g., Health Care for Homeless Veterans, HCHV), transitional housing (e.g., Grant Per Diem programs, GPD), and permanent supportive housing (Housing and Urban Development – VA Supportive Housing, HUD-VASH) programs.^19^ There are national initiatives that tailor primary care services for a subsegment of high-risk patients experiencing homelessness (Homeless Patient Aligned Care Team, HPACT)^20^ and that have provided over 100,000 tablets for video visits (Digital Divide consult).^21, 22^ Early in the pandemic, homeless-experienced veterans were found to use video visits less than housed veterans,^6^ but a subsequent study showed a higher overall rate of video care driven by homeless-experienced veterans’ higher usage of video for mental health care.^23^

While some research on tele-mental health services for veterans experiencing homelessness exists,^24, 25^ there remains a knowledge gap on the extent to which primary care services are delivered via telehealth. It is unknown whether these VA initiatives successfully engaged persons experiencing homelessness in primary care and characteristics of these telehealth users. It is possible that vulnerable populations, especially homeless-experienced persons, may have been left behind as telehealth expanded broadly. Alternatively, robust supportive services and available telehealth options may ensure equitable access to primary care. This national VA study aimed to examine the extent to which homeless-experienced veterans used telehealth services in primary care before and after the onset of the COVID-19 pandemic and characterized such users.

## METHODS

### Study Design and Cohort

This retrospective cohort study examined use of various telehealth modalities (phone, video, secure messaging) among 394,731 VA primary care patients with a diagnosis of homelessness between March 16, 2019, and March 15, 2022. We included three study years (0 = 1 year before COVID-19 onset, 1 = 1 year after COVID-19 onset, 2 = 2 years after COVID-19 onset). For each study year, we identified a cohort of patients who had at least one VA primary care visit and a diagnosis of homelessness within the study year. Homelessness was identified through International Classification of Diseases (ICD-10) codes Z59.0x, Z59.1x, Z59.8x, and Z59.9x.^26^ Study variables were extracted from VA administrative and electronic health records in the VA Corporate Data Warehouse. This quality improvement effort was deemed exempt from review by the VA Greater Los Angeles Healthcare System’s Institutional Review Board.

### Main Measures

The outcome measures were cohort patients having at least one video visit, one phone visit, or one secure message (My HealtheVet) within a study year. We examined the following patient characteristics which have been associated with differential telehealth use in prior research:^3–8^ age (18–44, 45–64, 65 +), birth sex (male, female), race-ethnicity (non-Hispanic White, non-Hispanic Black, Hispanic, non-Hispanic other, unknown race-ethnicity), Charlson Comorbidity Index (CCI) (0 = no comorbidity, 1 = one comorbidity, 2 +  = two or more comorbidities).^27^ Since VA homeless and mental health services (hereafter, “supportive services”) may facilitate telehealth adoption, we also examined the following: assignment to homeless-tailored primary care (HPACT) (yes/no),^20^ received supportive services from Health Care for Homeless Veterans (HCHV) (yes/no), Grant Per Diem (GPD) programs (yes/no), Housing and Urban Development – VA Supportive Housing (HUD-VASH) (yes/no), and Mental Health Intensive Case Management (MHICM, VA’s Assertive Community Treatment program) (yes/no).^19^

### Statistical Analysis

We conducted univariate (descriptive) analyses for all study variables by study year. We also conducted bivariate analyses and reported unadjusted percent telehealth use (video, phone, secure messaging) for each study variable by study year. For multivariable analyses, we conducted three logistic regression analyses (one for each outcome measure), adjusting for patient characteristics and VA supportive service variables. Models included fixed effects for each study year to account for secular trends, as well as adjusted for multiple observations of individuals over time. We reported odds ratios and 95% confidence intervals, as well as predicted probabilities (adjusted percent) of telehealth use while holding all covariates at their means.

## RESULTS

The study included 1,430,729 VA primary care visits (*n* = 218,127 patients) nationally 1 year prior to the COVID-19 pandemic; however, the numbers decreased slightly to 1,310,658 visits (*n* = 189,168 patients) 1 year after and 1,326,722 visits (*n* = 195,968 patients) 2 years after pandemic onset. Table 1 illustrates the descriptive data (unadjusted percent and sample size) for each study variable by study year. Patient characteristics and VA supportive service utilization remained stable over time. Compared to pre-pandemic, video and phone use among patients increased substantially 1 year after (video: 1.37% vs. 22.29%, phone: 60.74% vs. 88.94%), with a slight decrease 2 years after pandemic onset (video: 20.56%, phone: 76.58%). Secure messaging was low but also increased after pandemic onset (1.57% (year 0) vs. 2.46% (year 1) vs. 2.63% (year 2)) (Table 1).

**Table 1:** Demographic and Study Variables by Study Year for Veterans Experiencing Homelessness

	1 year before COVID onset 3/16/2019–3/15/2020 *n* = 218,127	1 year after COVID onset 3/16/2020–3/15/2021 *n* = 189,168	2 years after COVID onset 3/16/2021–3/15/2022 *n* = 195,968
Patient characteristics			
Age (mean, SD)	55.61 (14.24)	56.04 (14.14)	56.44 (14.41)
Age category % (*n*)			
18–44	22.85 (49,836)	22.38 (42,334)	22.84 (44,765)
45–64	51.48 (112,287)	50.29 (95,124)	46.83 (91,767)
65–74	19.23 (41,946)	20.96 (39,655)	23.27 (45,598)
75 +	6.44 (14,058)	6.37 (12,055)	7.06 (13,838)
Birth sex % (*n*)			
Male	88.03 (192,007)	88.00 (166,462)	87.46 (171,388)
Female	11.97 (26,120)	12.00 (22,706)	12.54 (24,580)
Race/ethnicity % (*n*)			
Non-Hispanic White	48.92 (106,701)	48.47 (91,688)	48.17 (94,388)
Non-Hispanic Black	37.14 (81,017)	37.17 (70,322)	37.12 (72,734)
Hispanic	6.85 (14,941)	6.83 (12,912)	6.72 (13,171)
Non-Hispanic other	2.53 (5511)	2.61 (4933)	2.62 (5135)
Unknown	4.56 (9957)	4.92 (9313)	5.38 (10,540)
Charlson Comorbidity Index % (*n*)			
0	52.24 (113,956)	50.84 (96,181)	53.99 (105,806)
1	19.5 (42,539)	19.57 (37,013)	19.37 (37,961)
2 +	28.26 (61,632)	29.59 (55,974)	26.64 (52,201)
VA supported services			
Assignment to Homeless-Tailored Primary Care Team, or Homeless Patient Aligned Care Team (HPACT)	6.39 (13,928)	5.72 (10,820)	5.08 (9948)
Outreach Services from Health Care for Homeless Veterans Program (HCHV)	46.45 (101,324)	43.16 (81,641)	46.38 (90,886)
Transitional Housing Program, or Grant Per Diem Program (GPD)	10.93 (23,840)	10.05 (19,002)	9.86 (19,328)
Permanent Supportive Housing Program, or Housing and Urban Development—VA Supportive Housing (HUD-VASH)	45.89 (100,095)	49.79 (94,195)	48.37 (94,783)
Mental Health Intensive Case Management (MHICM)	2.05 (4476)	2.41 (4551)	2.15 (4213)
Study outcome measures			
Used video	1.37 (2988)	22.29 (42,170)	20.56 (40,291)
Used phone	60.74 (132,496)	88.94 (168,246)	76.58 (150,063)
Used secure messaging	1.57 (3419)	2.46 (4645)	2.63 (5152)

Table 2 illustrates unadjusted percent telehealth use (video, phone, secure messaging) for each study variable during the most recent (year 2) data. Compared to the youngest age group (18–44 years old), 65 + -year-old patients had lower video use (14.15% vs. 29.35%) and secure messaging (1.47% vs. 3.91%). Conversely, 65 + -year-old patients had higher phone use (79.96%) compared to the youngest patients (71.91%). Compared to men, women had higher video use (32.57% vs. 18.84%), phone use (80.39% vs. 76.03%), and secure messaging (6.03% vs. 2.14%). Compared to non-Hispanic Whites, non-Hispanic Blacks had higher video use (22.22% vs. 18.43%), but lower phone use (75.74% vs. 77.37%) and secure messaging (2.41% vs. 2.79%). Patients experiencing homelessness who were assigned to homeless-tailored primary care (HPACT) had lower telehealth use for all three modalities: video, phone, and secure messaging (Table 2) than those assigned to usual primary care. Similarly, patients who used any other VA supportive services, except those who had intensive mental health case management (MHICM), had lower telehealth use for all three modalities (Table 2).

**Table 2:** Unadjusted Telehealth Use (Percent) by Demographic and Study Variables for Veterans Experiencing Homelessness (2 Years After COVID-19 Onset)

	Used video % (*n*)	Used phone % (*n*)	Used secure messaging % (*n*)
Patient characteristics			
Age category			
18–44	29.35 (13,139)	71.91 (32,190)	3.91 (1751)
45–64	20.42 (18,743)	76.66 (70,347)	2.75 (2525)
65 +	14.15 (8409)	79.96 (47,526)	1.47 (876)
Birth sex			
Male	18.84 (32,285)	76.03 (130,302)	2.14 (3670)
Female	32.57 (8006)	80.39 (18,761)	6.03 (1482)
Race/ethnicity			
Non-Hispanic White	18.43 (17,398)	77.37 (73,024)	2.79 (2638)
Non-Hispanic Black	22.22 (16,165)	75.74 (55,088)	2.41 (1752)
Hispanic	26.06 (3433)	77.28 (10,179)	3.09 (407)
Non-Hispanic other	20.10 (1032)	75.81 (3893)	2.67 (137)
Unknown	21.47 (2263)	74.75 (7879)	2.07 (218)
Charlson Comorbidity Index (CCI)			
0	21.59 (22,845)	71.31 (75,455)	2.59 (2741)
1	19.78 (7509)	79.97 (30,357)	2.85 (1082)
2 +	19.04 (9937)	84.77 (44,251)	2.55 (1329)
VA supported services			
Assignment to Homeless-Tailored Primary Care Team, Homeless Patient Aligned Care Team (HPACT)			
Yes	13.76 (1369)	73.06 (7268)	1.41 (140)
No	20.92 (38,922)	76.76 (142,795)	2.69 (5012)
Outreach Services from Health Care for Homeless Veterans Program (HCHV)			
Yes	20.57 (18,696)	75.70 (68,800)	2.52 (2294)
No	20.55 (21,595)	77.33 (81,263)	2.72 (2858)
Transitional Housing Program, or Grant Per Diem Program (GPD)			
Yes	18.85 (3643)	74.81 (14,460)	1.83 (353)
No	20.75 (36,648)	76.77 (135,603)	2.72 (4799)
Permanent Supportive Housing Program, or Housing and Urban Development—VA Supportive Housing (HUD-VASH)			
Yes	18.81 (17,829)	73.88 (70,025)	2.39 (2270)
No	22.20 (22,462)	79.10 (80,038)	2.85 (2882)
Mental Health Intensive Case Management (MHICM)			
Yes	19.63 (827)	79.33 (3342)	1.99 (84)
No	20.58 (39,464)	76.51 (146,721)	2.64 (5068)

Similar to unadjusted results, Table 3 illustrates the adjusted odds ratios (OR) and 95% confidence intervals (CI) for video use, phone use, and secure messaging use among cohort patients, adjusting for all study covariates. Compared to the youngest age group (18–44 years old), older patients were less likely to use video (45–64 years old: OR = 0.66, CI: 0.65–0.68; 65 + years old: OR = 0.43, CI: 0.42–0.44) and secure messaging (45–64 years old: OR = 0.77, CI: 0.74–0.81; 65 + years old: OR = 0.41, CI: 0.38–0.43), but more likely to use phone (45–64 years old: OR = 1.15, CI: 1.13–1.17; 65 + years old: OR = 1.19, CI: 1.17–1.22). Compared to men, women were more likely to use video (OR = 1.74, CI: 1.70–1.78), phone (OR = 1.46, CI: 1.43–1.49), and secure messaging (OR = 2.59, CI: 2.47–2.71). Compared to non-Hispanic Whites, non-Hispanic Blacks were more likely to use video (OR = 1.14, CI: 1.12–1.16), but less likely to use phone (OR = 0.92, CI: 0.91–0.93) and secure messaging (OR = 0.77, CI: 0.74–0.80). Compared to non-Hispanic Whites, Hispanics were more likely to use video (OR = 1.34, CI: 1.30–1.38) but less likely to use secure messaging (OR = 0.90, CI: 0.84–0.97). Compared to patients with no chronic comorbidity, patients with 1 or 2 + chronic comorbidities were more likely to use all three types of telehealth modalities (Table 3).

**Table 3:** Predictors of Telehealth Use (Video, Phone, Secure Messaging) for Veterans Experiencing Homelessness

	Used video Odds ratio (95% CI)	Used phone Odds ratio (95% CI)	Used secure messaging Odds ratio (95% CI)
Patient characteristics			
Age category (ref = 18–44)			
45–64	0.66 (0.65–0.68)*	1.15 (1.13–1.17)*	0.77 (0.74–0.81)*
65 +	0.43 (0.42–0.44)*	1.19 (1.17–1.22)*	0.41 (0.38–0.43)*
Birth sex			
Female	1.74 (1.70–1.78)*	1.46 (1.43–1.49)*	2.59 (2.47–2.71)*
Race/ethnicity (ref = NH White)			
Non-Hispanic Black	1.14 (1.12–1.16)*	0.92 (0.91–0.93)*	0.77 (0.74–0.80)*
Hispanic	1.34 (1.30–1.38)*	0.98 (0.96–1.01)	0.90 (0.84–0.97)*
Non-Hispanic other	1.04 (0.98–1.10)	0.89 (0.86–0.93)*	0.77 (0.68–0.88)*
Unknown	1.11 (1.07–1.16)*	0.85 (0.83–0.88)*	0.73 (0.66–0.81)*
Charlson Comorbidity Index (ref = 0)			
1	1.08 (1.05–1.10)*	1.58 (1.55–1.60)*	1.35 (1.29–1.42)*
2 +	1.16 (1.14–1.19)*	2.23 (2.19–2.27)*	1.47 (1.40–1.55)*
VA supported services			
Assignment to Homeless-Tailored Primary Care Team, Homeless Patient Aligned Care Team (HPACT)	0.68 (0.66–0.71)*	0.82 (0.80–0.84)*	0.59 (0.52–0.66)*
Outreach Services from Health Care for Homeless Veterans Program (HCHV)	0.90 (0.88–0.91)*	0.94 (0.93–0.96)*	0.86 (0.83–0.90)*
Transitional Housing Program, or Grant Per Diem Program (GPD)	0.98 (0.96–1.01)	0.98 (0.96–1.00)	0.80 (0.74–0.86)*
Permanent Supportive Housing Program, or Housing and Urban Development—VA Supportive Housing (HUD-VASH)	0.79 (0.78–0.81)*	0.76 (0.75–0.77)*	0.85 (0.82–0.88)*
Mental Health Intensive Case Management (MHICM)	0.99 (0.94–1.05)	1.26 (1.19–1.32)*	0.60 (0.51–0.70)*
COVID year onset (ref = 1 yr before)			
1 year after COVID onset	21.66 (20.86–22.49)*	5.43 (5.33–5.52)*	1.59 (1.53–1.66)*
2 years after COVID onset	19.6 (18.90–20.38)*	2.20 (2.17–2.23)*	1.74 (1.67–1.81)*

Logistic regression analysis was conducted for each telehealth modality (video, phone, secure messaging) after adjusting for study variables, study year, and repeated observations (i.e., patient clustering effect)

^*^p < .001

Primary care patients assigned to HPACT were less likely to use video (OR = 0.68, CI: 0.66–0.71), phone (OR = 0.82, CI: 0.80–0.84), and secure messaging (OR = 0.59, CI: 0.52–0.66). Similarly, patients who used HCHV and HUD-VASH services were less likely to use video, respectively (OR = 0.90, CI: 0.88–0.91; OR = 0.79, CI: 0.78–0.81), phone (OR = 0.94, CI: 0.93–0.96; OR = 0.76, CI: 0.75–0.77), and secure messaging (OR = 0.86, CI: 0.83–0.90; OR = 0.85, CI: 0.82–0.88). While there were no differences in video use, GPD (OR = 0.80, CI: 0.74–0.86) and MHICM users (OR = 0.60, CI: 0.51–0.70) were less likely to use secure messaging than their counterparts. However, MHICM users were more likely to use phone visits as part of their intensive case management (OR = 1.26, CI: 1.19–1.32).

Overall, fully adjusted models showed nearly a 22-fold increase in video visits for 1 year after (CI: 20.86–22.49) and 20-fold increase 2 years after pandemic onset (CI: 18.90–20.38), compared to the baseline year (Table 3). Phone visits increased fivefold (CI: 5.33–5.52) and twofold (CI: 2.17–2.23) in years 1 and 2, respectively, while secure messages increased 60% (CI: 1.53–1.66) and 70% (CI: 1.67–1.81), compared to pre-pandemic. The Appendix illustrates adjusted percent telehealth use (video, phone, secure messaging) for each study variable during the most recent study year.

## DISCUSSION

Despite a Digital Divide and low pre-pandemic use, telehealth in VA primary care increased significantly among veterans experiencing homelessness during the COVID-19 pandemic. To our knowledge, this is one of the largest studies to examine nearly 400,000 homeless-experienced persons, in over 4 million primary care visits, during a 3-year period that encompassed an unprecedented national disaster that necessitated telehealth adoption. Our study cohort was observed to increase use of video and phone visits at rates comparable to the general VA population.^17^ Post-pandemic, we found that 1 in 5 veterans experiencing homelessness participated in video visits, and the majority contacted their primary care teams by phone. High telehealth use was maintained beyond the first year of COVID-19 and as in-person primary care services returned. Findings were stable when we adjusted for patient factors that may affect telehealth use and show potential for virtual primary care use among a population severely affected by digital and other access barriers.^11, 13^ Secure messaging rates also increased to albeit a much lesser extent—only used by 2% of homeless-experienced veterans. Consistent with prior research,^28^ low secure messaging use may suggest that this primary care modality may not be preferred by persons experiencing homelessness or may have more barriers to adoption. It may be more difficult for veterans experiencing homelessness to use VA secure messaging, because secure messaging requires a formal enrollment process involving identity verification, which is not required for video and phone visits. Contextual (e.g., provider variation in telehealth adoption) and patient-level factors (e.g., patient digital health literacy and preference) that support or impede telehealth use among persons experiencing homelessness will need further exploration to ensure equitable access to primary care.

Our study of veterans experiencing homelessness found that telehealth usage patterns largely mirror that of the larger VA population. While homeless-experienced veterans tended to be younger, women, Black, and with fewer comorbidities^29^ than housed veterans, we still observed disproportionately higher use among those who were young, women, racial-ethnic minorities, and with multiple comorbidities. In our homeless-experienced cohort, we saw known age-related disparities in telehealth use^30^ over time, specifically 28% of 18–44-year-olds versus 15% of 65 + -year-olds used video visits (Appendix). We again found that women were open to^31^ and thus engaged with telehealth at higher rates than men, specifically 29% of women versus 19% of men used video visits and 6% of women versus 2% of men used secure messaging. Research on racial-ethnic disparities in telehealth use has been mixed;^4–8^ however, we did not see lower rates of telehealth use among racial-ethnic minorities in our cohort, specifically 19%, 22%, and 24% of White, Black, and Hispanic patients, respectively, used video visits. Finally, we observed that telehealth, especially phone visits, is helpful care management tool used by homeless-experienced veterans with multiple chronic comorbidities,^32^ specifically 71% with no comorbidity versus 85% with 2 + comorbidities used phone visits. Young, women, racial-ethnic minority, and high comorbidity veterans had higher video uptake proportionately, suggesting that telehealth may address access disparities among these homeless-experienced patient groups.

Interventions may be needed to increase telehealth uptake among patients assigned to homeless-tailored primary care (HPACT) clinics or who receive certain VA supportive services. Our study found that 15% of cohort patients assigned to HPACT versus 21% of those who received VA primary care elsewhere used video visits (Appendix). Even though HPACT selectively serves the most vulnerable patients experiencing homelessness,^20^ which likely contributes to this disparity, there may be room for implementation approaches that help increase adoption of virtual care among HPACT patients. Permanent supportive housing (HUD-VASH) is another setting that we could continue to target for telehealth interventions,^22^ since 18% of HUD-VASH users versus 22% of non-users used video visits. While HPACT or HUD-VASH users may be accustomed to in-person contact as required by certain supportive services (e.g., clothing distribution, medication delivery), it is worth exploring how best to optimize hybrid in-person and telehealth care to ensure equitable primary care access for the highly vulnerable veterans who use these services. On the other hand, video use was the same for veterans experiencing homelessness who participate in Mental Health Intensive Case Management (MHICM) and Transitional Housing (GPD) services, with minor differences in use of phone and secure messaging. Taking a positive deviance approach^33^ to study how MHICM and GPD programs successfully encourage telehealth use will likely inform best practices on engaging persons experiencing homelessness in virtual care.

While our national scope and large sample size is a clear strength in this study, limitations still apply. First, we used ICD-10 diagnostic codes to define our cohort of veterans experiencing homelessness. Our cohort excluded patients with homeless experiences who did not receive diagnostic coding, due to lack of standardized screening and coding of housing status, or in cases where informal supportive services were offered.^34^ Nonetheless, this cohort represents a patient population that willingly engages with VA services and can exercise preference for in-person versus virtual care. Second, administrative studies can be affected by variable telehealth service coding,^35^ where telehealth visits may be miscoded early on but hopefully remedied later during the pandemic. Third, our analyses did not include information on where patients lived or whether they had access to high-speed/broadband internet. Most (84%) of our homeless-experienced cohort resided in urban areas, which generally have adequate internet coverage areas,^36^ so this would not have likely changed our conclusions. Additionally, our administrative data sources did not contain patient-reported information on digital health literacy and preference, nor did we have information on provider variation in telehealth delivery for homeless-experienced veterans. Finally, since we did not analyze VA-funded services in the community, findings may or may not generalize to care provided by non-VA healthcare systems for veterans experiencing homelessness. However, prior studies show that the large majority of primary care services are delivered to veterans by the VA.^37^

The VA and other healthcare systems should continue to monitor use of and consider how best to incorporate telehealth services into primary care for patients experiencing homelessness. Ensuring equitable access to telehealth will help healthcare systems to engage its most vulnerable patients using all available modalities. Greater choice in how to access primary care services may in turn reduce utilization of acute care services (e.g., emergency visits, hospitalizations),^38^ as well as mitigate other disparities (e.g., racial-ethnic) in health and health care. Since patient characteristics remain immutable, identifying and targeting organizational characteristics (e.g., supportive housing users) that predict telehealth use for improvement may be key to increasing adoption among VA primary care patients experiencing homelessness.

### Supplementary information

ESM 1 (DOCX 18.3 kb)

## Data Availability

Under the Health Insurance Portability and Accountability Act (HIPAA), the dataset used in this study cannot be shared publicly because it contains patient-level Protected Health Information/Personally Identifiable Information (PHI/PII) from the Veterans Health Administration. To gain access to this data, interested researchers must complete credentialing to conduct VA research, as well as data use agreements with the Primary Care Analytics Team (pcat@va.gov) and other relevant VHA data owners.
