# Supplementary material for: Characteristics of Veterans Experiencing Homelessness using Telehealth for Primary Care Before and After COVID-19 Pandemic Onset
Source: J Gen Intern Med. 2024 Jan 22;39(Suppl 1):53–9. doi: 10.1007/s11606-023-08462-3 (PMC10937850; doi:10.1007/s11606-023-08462-3)
Supplement: Supplementary file 1 — (DOCX 18.3 kb) [file 11606_2023_8462_MOESM1_ESM.docx]

| Appendix 1. Adjusted Percent of Telehealth Use for Veterans Experiencing Homelessness (two-years after COVID-19 onset). | | | |
| --- | --- | --- | --- |
|  | Used Video  Percent (95% CI) | Used Phone  Percent (95% CI) | Used Secure Messaging  Percent (95% CI) |
| **Patient Characteristics** | | | |
| Age category |  |  |  |
| 18-44 | 28.10 (27.74-28.46) | 74.71 (74.42-75.01) | 3.67 (3.51-3.82) |
| 45-64 | 20.71 (20.48-20.94) | 77.25 (77.04-77.47) | 2.86 (2.77-2.96) |
| 65+ | 14.52 (14.29-14.76) | 77.81 (77.54-78.07) | 1.53 (1.46-1.61) |
| Birth Sex |  |  |  |
| Male | 19.39 (19.21-19.57) | 75.97 (75.77-76.16) | 2.22 (2.16-2.29) |
| Female | 29.31 (28.87-29.75) | 82.01 (81.71-82.32) | 5.53 (5.30-5.76) |
| Race/Ethnicity |  |  |  |
| Non-Hispanic White | 19.47 (19.26-19.69) | 77.53 (77.31-77.75) | 3.00 (2.90-3.09) |
| Non-Hispanic Black | 21.56 (21.31-21.81) | 76.06 (75.82-76.30) | 2.33 (2.34-2.42) |
| Hispanic | 24.29 (23.76-24.82) | 77.19 (76.75-77.63) | 2.72 (2.53-2.91) |
| Non-Hispanic Other | 20.08 (19.29-20.88) | 75.54 (74.83-76.25) | 2.33 (2.05-2.62) |
| Unknown | 21.17 (20.59-21.76) | 74.74 (74.20-75.27) | 2.22 (2.01-2.44) |
| Charlson Comorbidity Index (CCI) |  |  |  |
| 0 | 19.85 (19.64-20.06) | 71.47 (71.23-71.71) | 2.29 (2.21-2.36) |
| 1 | 21.02 (20.70-21.34) | 79.73 (79.45-80.00) | 3.06 (2.93-3.19) |
| 2+ | 22.30 (22.00-22.61) | 84.76 (84.54-84.97) | 3.31 (3.17-3.45) |
| **VA Supported Services** | | | |
| Assignment to Homeless-Tailored Primary Care Team, or Homeless Patient Aligned Care Team (HPACT) |  |  |  |
| Yes | 15.51 (14.97-16.05) | 73.35 (72.82-73.88) | 1.61 (1.42-1.81) |
| No | 21.00 (20.81-21.17) | 76.99 (76.80-77.17) | 2.72 (2.64-2.79) |
| Outreach Services from Health Care for Homeless Veterans Program (HCHV) |  |  |  |
| Yes | 19.78 (19.55-20.00) | 76.24 (76.02-76.46) | 2.36 (2.38-2.54) |
| No | 21.50 (21.28-21.72) | 77.22 (77.01-77.42) | 2.84 (2.75-2.92) |
| Transitional Housing Program, or Grant Per Diem Program (GPD) |  |  |  |
| Yes | 20.44 (20.01-20.86) | 76.45 (76.62-77.00) | 2.18 (2.03-2.33) |
| No | 20.72 (20.54-20.90) | 76.80 (76.08-76.83) | 2.71 (2.64-2.78) |
| Permanent Supportive Housing Program, Housing and Urban Development - VA Supported Housing (HUD-VASH) |  |  |  |
| Yes | 18.80 (18.59-19.02) | 74.23 (73.99-74.46) | 2.45 (2.36-2.53) |
| No | 22.46 (22.23-22.69) | 79.10 (78.90-79.30) | 2.87 (2.78-2.29) |
| Mental Health Intensive Case Management (MHICM) |  |  |  |
| Yes | 20.64 (19.78-21.49) | 80.42 (79.69-81.14) | 1.64 (1.38-1.88) |
| No | 20.69 (20.52-20.87) | 76.68 (76.50-76.87) | 2.69 (2.62-2.76) |

* Predicted probabilities for each telehealth modality (video, phone, secure messaging) after adjusting for study variables, study year, and repeated observations (i.e., patient clustering effect).
